# Analysis of endometrial lavage microbiota reveals an increased relative abundance of the plastic-degrading bacteria *Bacillus pseudofirmus* and *Stenotrophomonas rhizophila* in women with endometrial cancer/endometrial hyperplasia

**DOI:** 10.3389/fcimb.2022.1031967

**Published:** 2022-11-09

**Authors:** Angel Chao, An-Shine Chao, Chiao-Yun Lin, Cindy Hsuan Weng, Ren-Chin Wu, Yuan-Ming Yeh, Shih-Sin Huang, Yun-Shien Lee, Chyong-Huey Lai, Huei-Jean Huang, Yun-Hsin Tang, Yu-Shan Lin, Chin-Jung Wang, Kai-Yun Wu

**Affiliations:** ^1^ Department of Obstetrics and Gynecology, Linkou Chang Gung Memorial Hospital and Chang Gung University College of Medicine, Taoyuan, Taiwan; ^2^ Gynecologic Cancer Research Center, Linkou Chang Gung Memorial Hospital, Taoyuan, Taiwan; ^3^ Department of Obstetrics and Gynecology, New Taipei Municipal Tu Cheng Hospital, New Taipei City, Taiwan; ^4^ Department of Pathology, Linkou Chang Gung Memorial Hospital and Chang Gung University College of Medicine, Taoyuan, Taiwan; ^5^ Genomic Medicine Research Core Laboratory, Chang Gung Memorial Hospital, Taoyuan, Taiwan; ^6^ Department of Biotechnology, Ming-Chuan University, Taoyuan, Taiwan

**Keywords:** microbiota, endometrial cancer, endometrial hyperplasia, plastics, environmental pollution

## Abstract

The pathogenic influences of uterine bacteria on endometrial carcinogenesis remain unclear. The aim of this pilot study was to compare the microbiota composition of endometrial lavage samples obtained from women with either endometrial hyperplasia (EH) or endometrial cancer (EC) *versus* those with benign uterine conditions. We hypothesized that specific microbiota signatures would distinguish between the two groups, possibly leading to the identification of bacterial species associated with endometrial tumorigenesis. A total of 35 endometrial lavage specimens (EH, n = 18; EC, n = 7; metastatic EC, n = 2; benign endometrial lesions, n = 8) were collected from 32 women who had undergone office hysteroscopy. Microbiota composition was determined by sequencing the V3−V4 region of 16S rRNA genes and results were validated by real-time qPCR in 46 patients with EC/EH and 13 control women. Surprisingly, we found that *Bacillus pseudofirmus* and *Stenotrophomonas rhizophila* – two plastic-degrading bacterial species – were over-represented in endometrial lavage specimens collected from patients with EC/EH. Using computational analysis, we found that the functional profile of endometrial microbiota in EC/EH was associated with fatty acid and amino acid metabolism. In summary, our hypothesis-generating data indicate that the plastic-degrading bacteria *Bacillus pseudofirmus* and *Stenotrophomonas rhizophila* are over-represented within the endometrial lavage microbiota of women with EC/EH living in Taiwan. Whether this may be related to plastic pollution deserves further investigation.

## Introduction

Despite decades of intense research, endometrial cancer (EC) remains a substantial public health problem in women (Lortet-Tieulent et al., 2018; Zhang et al., 2019). While the underlying reasons are multifaceted (Felix and Brinton, 2018), growing evidence suggests that environmental pollution can be linked with an increased risk of EC (Dunnick et al., 2015; Mallozzi et al., 2017). Interestingly, exposure to estrogen-mimicking endocrine disruptors – including certain preservatives and industrial plasticizers that can be biologically active at extremely low levels (Hiroi et al., 2004; Chou et al., 2017) – has been related to endometrial carcinogenesis (Yaguchi, 2019; Wen et al., 2020). Furthermore, the ability of plastic particles to accumulate in human tissues, including the reproductive tract, is well established (Zlatnik, 2016; Waring et al., 2018).

Prior studies using molecular techniques have reported that the human endometrium has a resident microbiota dominated by *Lactobacillus*, *Pseudomonas*, and *Acinetobacter* (Chen et al., 2017). In recent years, there have been attempts to investigate the role of uterine dysbiosis in various intrauterine diseases (Baker et al., 2017). Interestingly, plastic pollution has the potential to affect human tissue microbiota (Velmurugan et al., 2017; Lu et al., 2019) either *via* direct toxic effects or by providing supplemental carbon sources (Lear et al., 2021). However, there are limited data available to determine possible links between uterine dysbiosis and endometrial tumorigenesis (Walther-Antonio et al., 2016; Chen et al., 2021; Lu et al., 2021).

Genetic sequencing of uterine lavage samples obtained *via* office hysteroscopy has recently emerged as a less invasive approach for real-time diagnosis and monitoring of EC (Chao et al., 2022). By taking advantage of this technique, the goal of this pilot study was to compare the microbiota composition of endometrial lavage samples obtained from Taiwanese women with either endometrial hyperplasia (EH) or EC *versus* those with benign uterine conditions. We hypothesized that specific microbiota signatures would distinguish between the two groups, possibly leading to the identification of bacterial species associated with endometrial tumorigenesis. Surprisingly, the key dysbiosis identified in endometrial lavage samples collected from women with EC/EH was an increased relative abundance of the two plastic-degrading bacteria *Bacillus pseudofirmus* and *Stenotrophomonas rhizophila* (Dela Torre et al., 2018; Wei et al., 2018; Danso et al., 2019; Atanasova et al., 2021).

## Materials and methods

### Participants

Women with recent abnormal uterine bleeding due to a suspected endometrial lesion (e.g., polyp, myoma, EH, or EC) who were referred for an office hysteroscopy (Salazar and Isaacson, 2018) were eligible, as were those with either a newly diagnosed or known EH/early EC who had undergone fertility-preserving treatment (Chao et al., 2011; Gunderson et al., 2012; Hubbs et al., 2013; Yang et al., 2020). The enrolment started in July 2020 and concluded in June 2021. Women with suspected endometritis and those unwilling to participate were excluded. Endometrial biopsies collected during office hysteroscopy were used for achieving a final pathological diagnosis. Ethical approval was granted by the Institutional Review Board (reference number: 202100083B0) of the Chang Gung Memorial Hospital, Taiwan. All women provided written informed consent before enrolment.

### Collection of endometrial lavage specimens

Office hysteroscopy was performed with the vaginoscopic technique (Vitale et al., 2020). Prior to endometrial biopsies, normal saline was instilled to provide distension and irrigation of the uterine cavity. Endometrial lavage samples (25 mL) obtained using a continuous-flow rigid hysteroscopy system (sheath diameter: 4 mm; Richard Wolf GmbH, Knittlingen, Germany) were collected in sterile tubes and centrifuged at 3200 rpm for 20 min at 4°C. Cell pellets were suspended in PBS, washed with a red blood cell lysis solution, incubated at room temperature for 30 min, and centrifuged at 3000 rpm for 10 min. After removal of the supernatant, pellets were stored -80°C until analysis (Nair et al., 2016).

### Bacterial DNA preparation, library construction, and sequencing

Bacterial DNA for microbiota analysis was extracted using a QiaAmp DNA Microbiome Kit (Qiagen, Hilden, Germany). A Qubit dsDNA High Sensitivity Assay (Thermo Fisher Scientific, Waltham, MA, USA) was used to determine the concentration and quality of purified DNA. The protocol for library construction and sequencing has been previously described in detail (Lin et al., 2020). In brief, a 16S rRNA gene amplicon library targeting the 16S rRNA V3−V4 region was initially constructed. Illumina adaptor overhang nucleotide sequences were subsequently added to gene-specific sequences. The 16S rRNA gene amplicon PCR primers were as follows: forward, 5’-TCGTCGGCAGCGTCAGATGTGTATAAGAGACAGCCTACGGGNGGCWGCAG-3’ and reverse: 5’-GTCTCGTGGGCTCGGAGATGTGTATAAGAGACAGGACTACHVGGGTATCTAATCC-3’.

### Bioinformatics analysis of amplicon library sequences

The details of bioinformatics analysis of amplicon library sequences have been published (Lin et al., 2020). Briefly, sequencing reads were initially de-multiplexed using the Miseq Reporter tool v2.6 (Illumina, San Diego, CA, USA) based on sample barcodes. The Usearch tool v11 (https://drive5.com/) was used for processing raw reads. A chimera removal step was implemented to ensure that any sequencing-related error was removed through the selection of qualified reads. Species richness and diversity were determined based on the number of bacterial species assigned by amplicon sequence variants (ASVs). We estimated richness using the Observed ASV and Chao1 indices. We also obtained measures of α-diversity – which focuses on variation within a community (e.g., Shannon index and Gini-Simpson index) and β-diversity – which quantifies dissimilarities between communities based on the principal component analysis plot constructed with unweighted UniFrac. Finally, we used LEfSe20 to identify the taxonomy of bacteria that were most likely to distinguish women with either EH or EC *versus* those with benign uterine conditions.

### Real time qPCR

The TaqMan gene expression assay (Applied Biosystems, Foster City, CA, USA) was used to quantify DNA from *Bacillus pseudofirmus* (Assay ID: ART2CTZ) and *Stenotrophomonas rhizophila* (Assay ID: APH6GAT). Data were normalized using 16S rRNA expression levels (Assay ID: Ba04930791_s1). When the fluorescent signal did not increase until 40 cycles, the sample was assigned an arbitrary value of 40. The -ΔΔCt was calculated according to the following formula: ΔCt (bacteria) − ΔCt (internal control). Correlation analyses of ΔCt values were performed to assess their reciprocal associations (Lin et al., 2020).

### Microbiota data analysis

ASV relative abundances (expressed as percentages) were log-transformed prior to further analyses (Lin et al., 2020). The metagenome content was predicted using the Phylogenetic Investigation of Communities by Reconstruction of Unobserved States (PICRUSt) and the Kyoto Encyclopedia of Genes and Genomes (KEGG) pathway. Data concerning the mid-vaginal microbiota of healthy non-pregnant women and the cervical microbiota of pregnant women were obtained from the Human Microbiome Project (HMP) (Human Microbiome Project C, 2012a; Human Microbiome Project C, 2012b) and the Integrative Human Microbiome Project (iHMP) (Integrative H.M.P.R.N.C, 2019), respectively. Statistical analyses were performed using R (http://www.r-project.org/), unless otherwise indicated.

## Results

A total of 35 endometrial lavage specimens (EH, n = 18; EC, n = 7; metastatic EC, n = 2; benign endometrial lesions, n = 8) were collected from 32 women who had undergone office hysteroscopy **(** **Supplementary Figure 1**; **Supplementary Table 1** **)**. For the purpose of analysis, samples were categorized as follows: patients with premalignant and malignant endometrial lesions (EC/EH; n = 27) *versus* control women (n = 8).

### Richness and diversity of endometrial lavage microbiota

**Figure 1:**
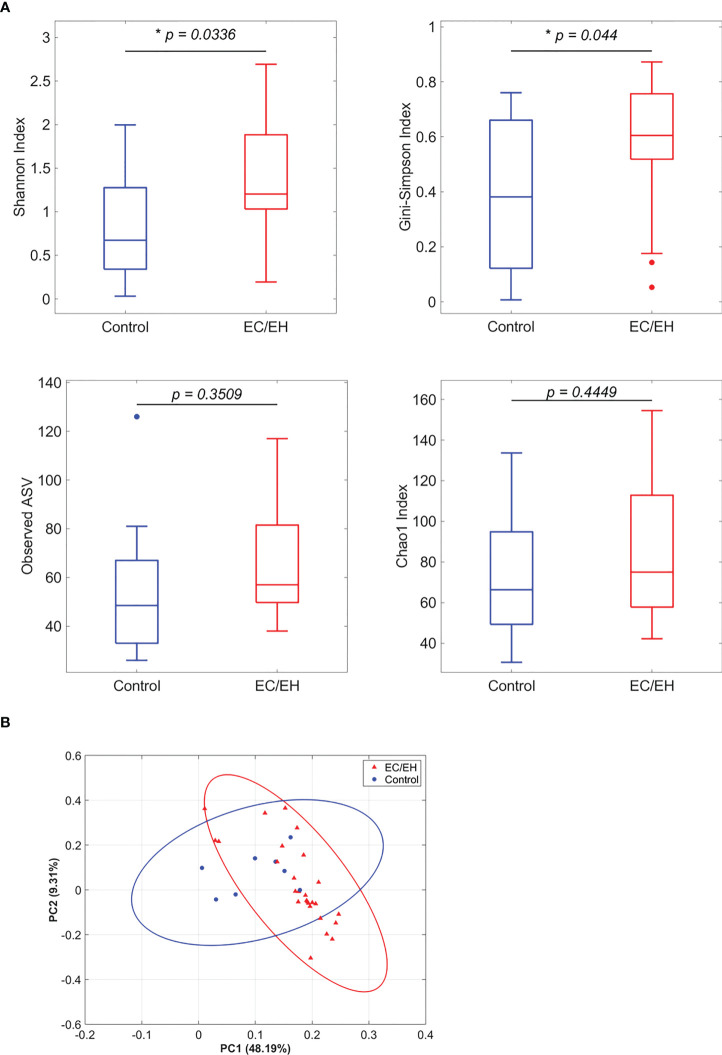
Analysis of endometrial lavage microbiota in patients with EC/EH *versus* control women. **(A)** Richness and diversity indices. **(B)** Principal component analysis based on unweighted UniFrac distances. “*” means statistically significant P<0.05.

Endometrial lavage bacterial microbiota was characterized using 16S rRNA V3−V4 gene sequence analysis. Patients with EC/EH did not differ from control women in terms of species richness (Observed ASV and Chao1 indices). However, the former group had a higher α-diversity compared with the latter (Shannon index, *P* = 0.034; Gini-Simpson index, *P* = 0.044; **Figure 1A**). While the two study groups did not differ significantly in terms of taxonomic β-diversity, there was a trend towards a higher concentration in the middle portion for patients with EC/EH **(** **Figure 1B** **)**.

### Composition of endometrial lavage microbiota

**Figure 2:**
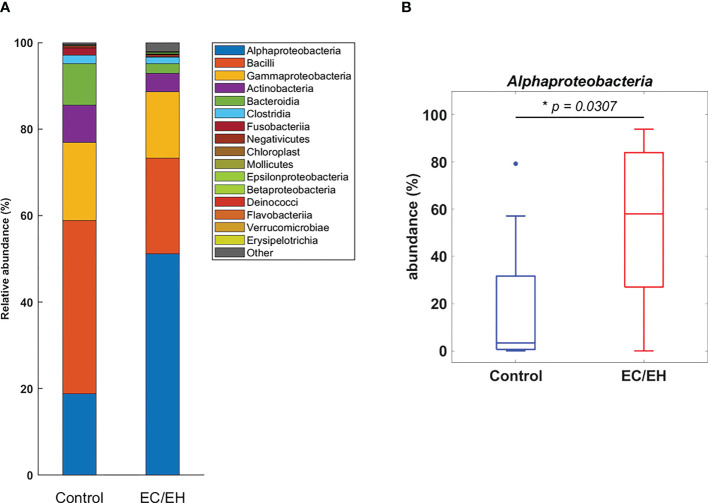
**(A)** Relative abundance of endometrial lavage bacteria in patients with EC/EH *versus* control women: taxonomic identification at the class level. **(B)** Box plots revealed a statistically significant intergroup difference in the relative abundance of the *Alphaproteobacteria* class. “*” means statistically significant P<0.05.

**Figure 3:**
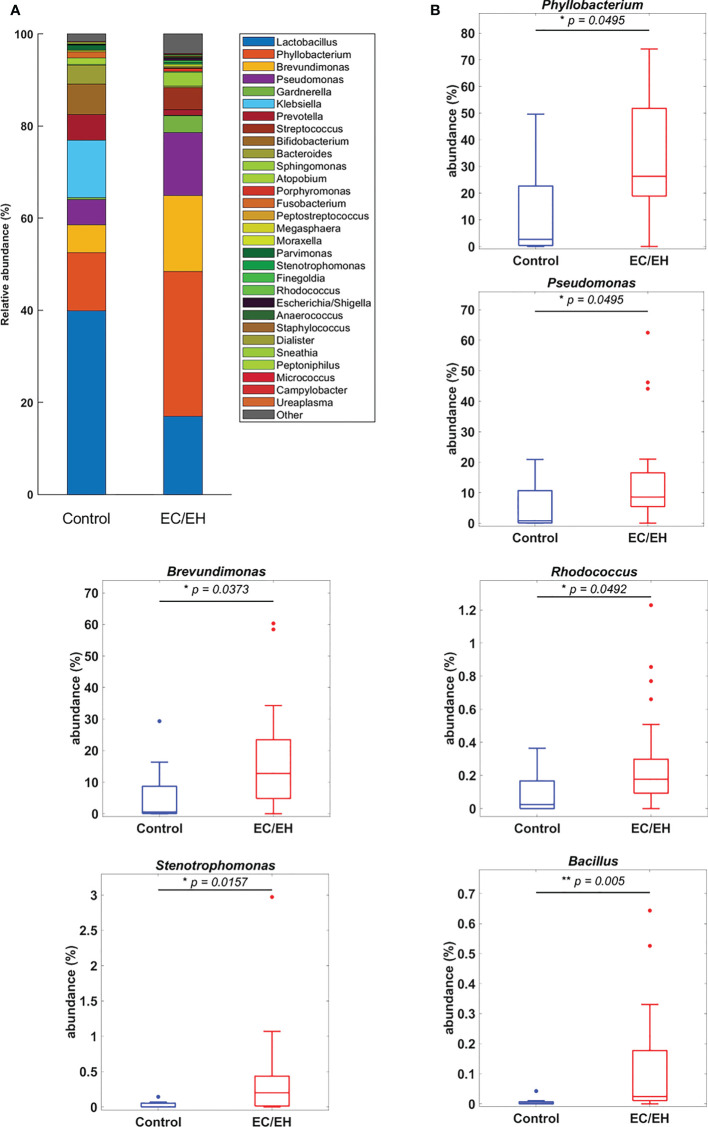
**(A)** Relative abundance of endometrial lavage bacteria in patients with EC/EH *versus* control women: taxonomic identification at the genus level. **(B)** Box plots revealed statistically significant intergroup differences in the relative abundance of the genera *Phyllobacterium*, *Pseudomonas*, *Brevundimonas*, *Rhodococcus*, *Stenotrophomonas*, and *Bacillus*. “*“ means statistically significant P<0.05, “**” P<0.01.

**Figure 4:**
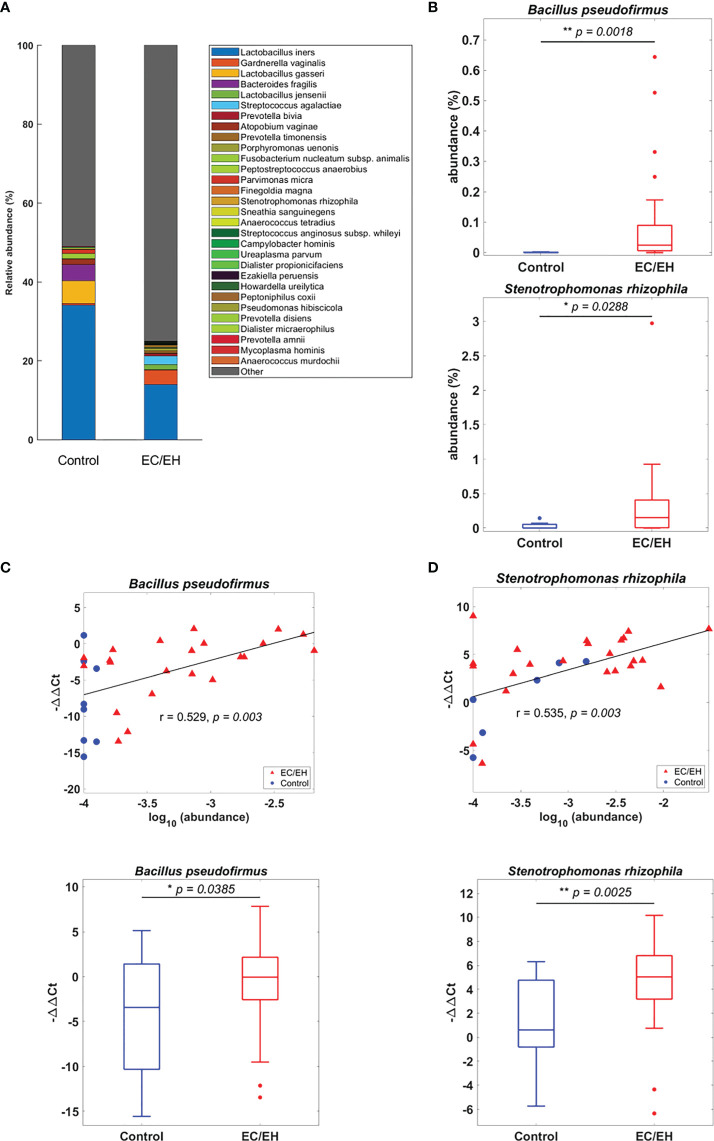
**(A)** Relative abundance of endometrial lavage bacteria in patients with EC/EH *versus* control women: taxonomic identification at the species level. **(B)** Box plots revealed statistically significant intergroup differences in the relative abundance of the species *Bacillus pseudofirmus* and *Stenotrophomonas rhizophila*. **(C)** The higher relative abundance of *Bacillus pseudofirmus* in patients with EC/EH was confirmed by the positive correlation between the results of gene sequencing (r = 0.529, *P* = 0.003; upper panel) and those of real-time qPCR (EC/EH samples, n = 54, control, n = 13, lower panel). **(D)** The higher relative abundance of *Stenotrophomonas rhizophila* in patients with EC/EH was confirmed by the positive correlation between the results of gene sequencing (r = 0.535, *P* = 0.003) and those of real-time qPCR (lower panel). “*“ means statistically significant P<0.05, “**” P<0.01.

The relative abundance of endometrial lavage bacteria was analyzed at three taxonomic ranks (class, genus, and species). At the class level, *Alphaproteobacteria* were more commonly represented in patients with EC/EH than in control women (*P* = 0.0307; **Figure 2**). At the genus level, the *Bacillus* (*P* = 0.005), *Stenotrophomonas* (*P* = 0.0157), *Phyllobacterium* (*P* = 0.0495), *Pseudomonas* (*P* = 0.0495), *Brevundimonas* (*P* = 0.0373), and *Rhodococcus* (*P* = 0.0492) genera were over-represented in patients with EC/EH **(** **Figure 3** **)**. Conversely, *Lactobacilli* and *Bifidobacterium* were under-represented. At the species level, we found that *Bacillus pseudofirmus* (*P* = 0.0018; **Figure 4A**) and *Stenotrophomonas rhizophila* (*P* = 0.0288; **Figure 4B**) were over-represented in patients with EC/EH. This finding was validated using real-time qPCR in a larger cohort of patients with EC/EH (54 lavage samples obtained from 46 patients) and control women (13 lavage samples obtained from 13 women; **Supplementary Table 1** **)**. The results confirmed the higher relative abundance of both *Bacillus pseudofirmus* **(** **Figure 4C** **)** and *Stenotrophomonas rhizophila* **(** **Figure 4D** **)** in the endometrial lavage microbiota of the former group **(** **Supplementary Table 2** **)**.

### Linear discriminant analysis of taxonomic profiles

**Figure 5:**
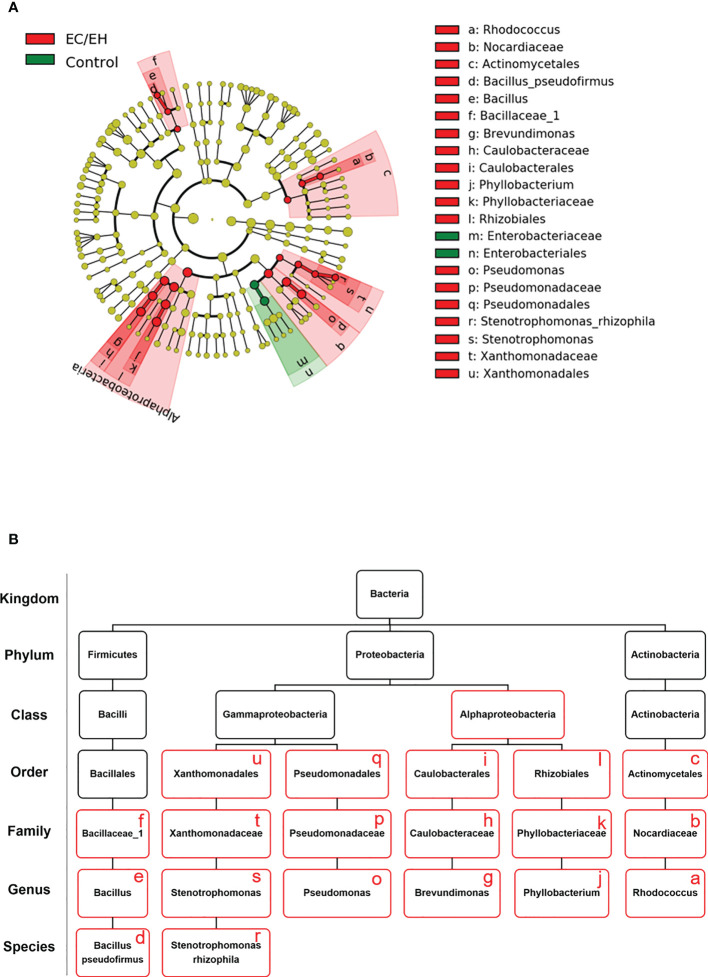
Taxonomic profiles of endometrial lavage microbiota in patients with EC/EH *versus* control women: results of linear discriminant analysis of effect size (LEfSe). **(A)** Cladogram plots were generated to visualize significantly enriched bacterial taxa in the two study groups. **(B)** Summary of bacterial taxa showing significant differences between patients with EC/EH *versus* control women (red boxes).

A linear discriminant analysis of effect size (LEfSe) was undertaken to compare the taxonomic profiles of endometrial lavage microbiota in patients with EC/EH *versus* control women **(** **Figure 5A** **)**. Significant intergroup differences were observed at different taxonomic ranks (class, family, genus, and species; **Figure 5B** **)**. This analysis consistently supported the differences in the relative abundance of *Bacillus pseudofirmus* and *Stenotrophomonas rhizophila* between the two study groups **(** **Supplementary Table 3** **)**.

### Functional profile of endometrial lavage microbiota

**Figure 6:**
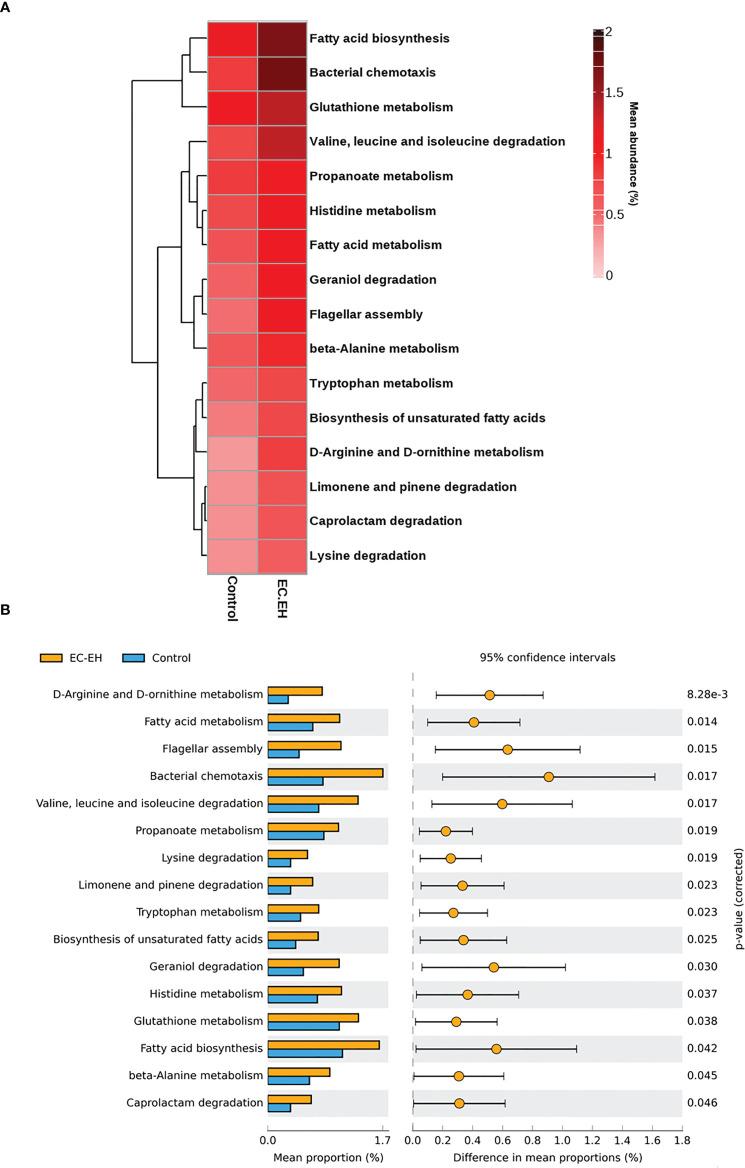
PICRUSt prediction of functional pathways in endometrial lavage microbiota of patients with EC/EH *versus* control women. **(A)** Relative abundance of different functional profiles. **(B)** Functional pathways upregulated in patients with with EC/EH.

The PICRUSt tool was implemented to predict the functional profile of endometrial lavage microbiota in patients with EC/EH. The results revealed an involvement of the following two pathways **(** **Figure 6** and **Supplementary Table 4** **)**: 1) fatty acid metabolism (including fatty acid biosynthesis and biosynthesis of unsaturated fatty acids; and 2) amino acid metabolism (including D-arginine and D-ornithine metabolism; valine, leucine, and isoleucine degradation; lysine degradation; tryptophan metabolism; histidine metabolism; glutathione metabolism; and beta-alanine metabolism).

### Validation by comparison between endometrial lavage and vaginal microbiota

**Figure 7:**
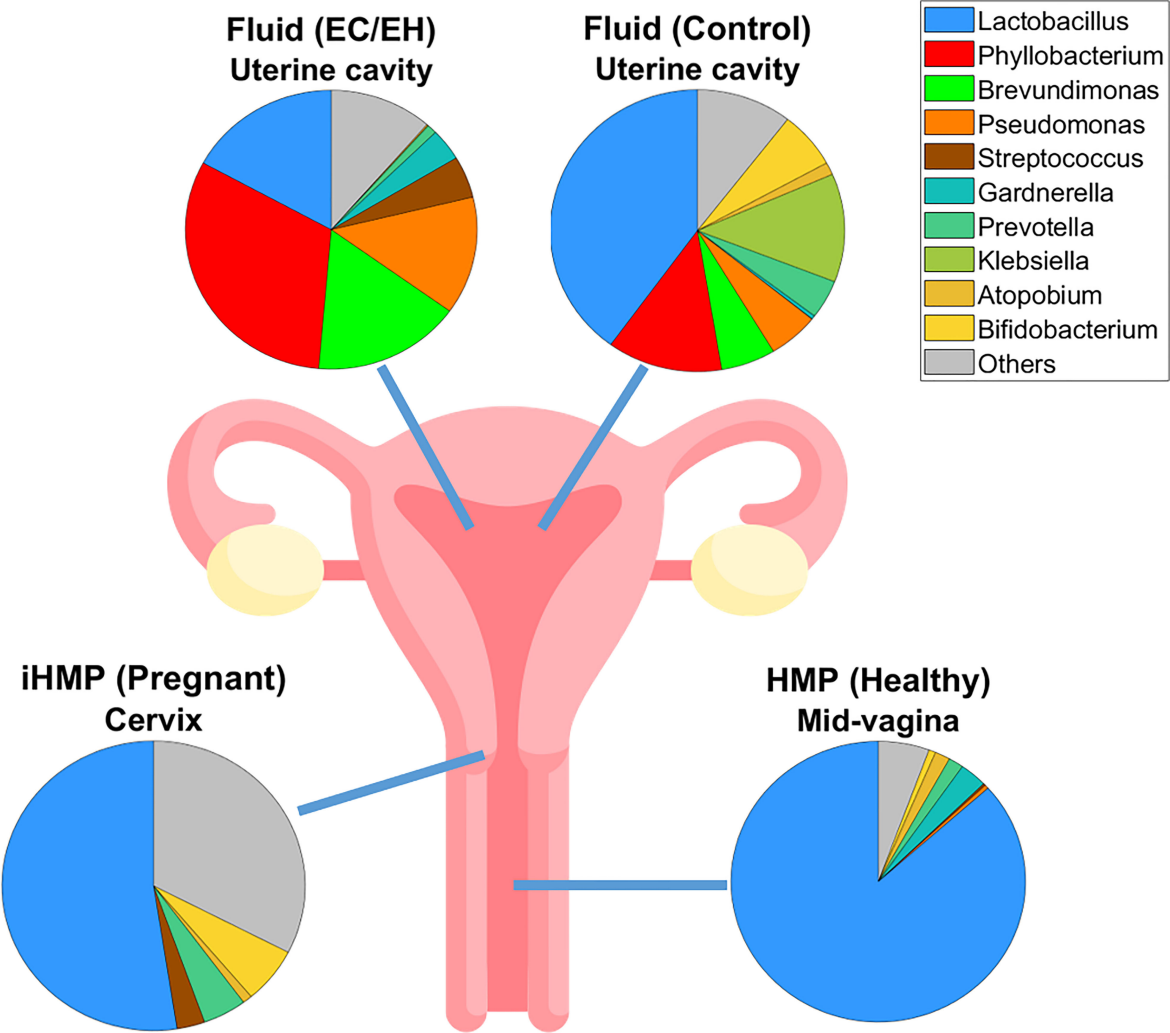
Pie plots showing the microbial compositions at the genus level in various levels of the female reproductive tract. A stepwise decrease in the relative abundance of the genus *Lactobacillus* was observed, as follows: 1) mid-vaginal microbiota of healthy non-pregnant women, 2) cervical microbiota of pregnant women, 3) endometrial lavage microbiota of women with benign endometrial lesions, and 4) endometrial lavage microbiota of women with EC/EH. Data on the mid-vaginal microbiota of healthy non-pregnant women and the cervical microbiota of pregnant women were obtained from the Human Microbiome Project (HMP) and the Integrative Human Microbiome Project (iHMP), respectively.

Finally, sequencing data were compared using the HMP and iHMP datasets (**Figure 7** and **Supplementary Table 5**). We found a stepwise decrease in the relative abundance of the genus *Lactobacillus* in the following microbiotas: 1) mid-vaginal microbiota of healthy non-pregnant women, 2) cervical microbiota of pregnant women, 3) endometrial lavage microbiota of women with benign endometrial lesions, and 4) endometrial lavage microbiota of women with EC/EH.

## Discussion

The novel finding of this pilot, hypothesis-generating study is that *Bacillus pseudofirmus* and *Stenotrophomonas rhizophila* – two plastic-degrading bacteria (Dela Torre et al., 2018; Wei et al., 2018; Danso et al., 2019; Atanasova et al., 2021) – are over-represented within the endometrial lavage microbiota of Taiwanese women with EC/EH. On the one hand, *Bacillus pseudofirmus* is a facultative aerobic alkaliphile bacterium characterized by the ability to degrade inert polyethylene-based plastics, including low-density polyethylene (LDPE) (Dela Torre et al., 2018; Atanasova et al., 2021). On the other hand, *Stenotrophomonas rhizophila* is not only an efficient degrader of polyvinyl alcohol (Wei et al., 2018) but can also remove heavy metals from contaminated water (Hagagg et al., 2020). We speculate that these two bacterial species may be enriched in the uterine microbiota of women with EC/EH as a result of an increased exposure to environmental pollutants. However, there is no published evidence that *Bacillus pseudofirmus* and/or *Stenotrophomonas rhizophila* can cause human infections and/or known disease conditions (Berg and Martinez, 2015; Mukherjee and Roy, 2016; Brooke et al., 2017).

This is, to our knowledge, the first study to demonstrate that these two bacterial species can colonize the human body in general and the uterine cavity in particular. While this was a surprising finding, we believe that the detection of *Bacillus pseudofirmus* and *Stenotrophomonas rhizophila* in uterine lavage fluid cannot be considered an artifact for at least two reasons. First, on analyzing bioinformatics data, we implemented a chimera removal step to ensure that any sequencing-related error was removed. Second, we successfully validated the over-representation of the two bacterial species using real-time qPCR in a larger cohort of patients with EC/EH. It is possible that the colonization of the uterine cavity by *Bacillus pseudofirmus* and *Stenotrophomonas rhizophila* in this patient group could reflect an interaction between the composition of local microbiota and other variables. An interaction exists when the relationship between an independent variable x (e.g., contact of the human body with a given bacterial species) and an outcome variable y (e.g., ability of the species to colonize the body after contact and/or causing a disease) varies according to the value of another covariate z (e.g., presence of plastic pollutants). On the one hand, it is plausible that previous studies that failed to detect *Bacillus pseudofirmus* and *Stenotrophomonas rhizophila* in the uterine microbiota might have been conducted in women not exposed to plastic pollution. On the other hand, if independently confirmed by future research, *Bacillus pseudofirmus* and *Stenotrophomonas rhizophila* may emerge as flagship species for plastic pollution in humans. Importantly, our results highlight the need to include uterine lavage fluid as target samples in studies exploring the impact of plastic contamination on women’s health. Both LDPE and certain heavy metals, which are degraded and removed by *Bacillus pseudofirmus* (Dela Torre et al., 2018; Atanasova et al., 2021) and *Stenotrophomonas rhizophila* (Hagagg et al., 2020), respectively, may act as estrogen-mimicking endocrine disruptors to promote endometrial carcinogenesis (Yang et al., 2011; Rzymski et al., 2015). Interestingly, Taiwan is characterized by a markedly high daily intake of estrogen-like pollutants (Lu et al., 2007).

While this pilot study is the first to link the presence of plastic-degrading bacteria in the uterine microbiota with the risk of EC/EH, the higher abundance of *Phyllobacterium* and *Rhodococcus* observed in our study is in accordance with the published literature (Lu et al., 2021). Collectively, these results support the robustness of our analysis. Using computational analysis, we found that the functional profile of endometrial lavage microbiota in EC/EH was associated with fatty acid and amino acid metabolism. Microbiota-derived fatty acids may promote tumorigenesis *via* chronic local inflammation and sustained immune reactions (Mirzaei et al., 2021), whereas amino acids can favor the survival of malignant cells under nutritional, oxidative, and genotoxic stress (Wei et al., 2020). Apart from the detection of *Bacillus pseudofirmus* and *Stenotrophomonas rhizophila*, we also observed that the probiotic bacteria *Lactobacillus* and *Bifidobacterium* were under-represented in the endometrial lavage of patients with EC/EH. This finding is in line with published data (Wieers et al., 2019) and may have contributed to the creation of a carcinogenic milieu through a decreased probiotic-derived production of antitumor molecules (Alizadehmohajer et al., 2020).

Several limitations of our study are worth noting. First, the question as to whether the detection of *Bacillus pseudofirmus* and *Stenotrophomonas rhizophila* in uterine lavage fluid is the result of a rapid transit rather than of an effective colonization remains unanswered. A longitudinal investigation with serial sampling of uterine lavage fluid may work to offer a solution to this conundrum. Second, an analysis of microbiota from other body sites in women with EC/EH would have been interesting; unfortunately, as we did not collect these samples for the purpose of the current study, we cannot provide these data. Third, we are currently unable to clarify whether the presence of *Bacillus pseudofirmus* and *Stenotrophomonas rhizophila* is the result of environmental contamination or rather it is environmental contamination that affects their behavior. Fourth, it would have been interesting to perform plastic particle extraction and quantification from the uterine fluid. Unfortunately, our laboratory is currently unable to run the analytical procedure to obtain these data. Finally, we acknowledge the possibility that medical procedures could have acted as a source of plastic contamination in patients with EC/EH.

## Conclusions

Our pilot data indicate that the plastic-degrading bacteria *Bacillus pseudofirmus* and *Stenotrophomonas rhizophila* are over-represented within the endometrial lavage microbiota of women in Taiwan with EC/EH. Despite the intrigue of connecting plastic pollution, uterine microbiota, and different endometrial disease phenotypes, evaluating the hypotheses outlined in our study will require more holistic approaches incorporating serial uterine lavage fluid sampling to identify whether findings are reproducible over time, as well as direct measures of plastic pollution to understand if the observed associations are truly driven by causation.

## Data availability statement

The datasets presented in this study can be found in online repositories. The names of the repository/repositories and accession number(s) can be found below: https://www.ncbi.nlm.nih.gov/, PRJNA843535.

## Ethics statement

Ethical approval was obtained from the local Institutional Review Board (reference number: 202100083B0) of Chang Gung Memorial Hospital, Taiwan. The patients/participants provided their written informed consent to participate in this study.

## Author contributions

AC, K-YW and A-SC: study concept and design; K-YW, C-W, Y-MY, S-SH, Y-SLee, Y-HT, H-JH, Y-SLin, C-HL, A-SC, and C-JW: data collection and interpretation; R-CW: pathological examinations; AC, C-YL, and K-YW: manuscript drafting; AC, A-SC, and C-YL: critical revision of the manuscript for important intellectual content. All authors contributed to the article and approved the submitted version.

## Funding

This study was financially supported by grants from the Ministry of Science and Technology (110-2314-B-182A-027-MY3) and the Chang Gung Medical Foundation (CIRPG3K0031/2 to AC and CMRPG3M0511 to K-YW).

## Acknowledgments

The authors wish to acknowledge Jung-Erh Yang and Chu-Chun Huang for their excellent technical assistance.

## Conflict of interest

The authors declare that the research was conducted in the absence of any commercial or financial relationships that could be construed as a potential conflict of interest.

## Publisher’s note

All claims expressed in this article are solely those of the authors and do not necessarily represent those of their affiliated organizations, or those of the publisher, the editors and the reviewers. Any product that may be evaluated in this article, or claim that may be made by its manufacturer, is not guaranteed or endorsed by the publisher.

## Supplementary material

Supplementary Table 1 Characteristics of uterine lavage samples analyzed in the study.

Supplementary Table 2 Relative abundance of *Bacillus pseudofirmus* and *Stenotrophomonas rhizophila* according to real-time qPCR results.

Supplementary Table 3 Abundance of taxa in patients with EC/EH (n = 27) and control women (n = 8).

Supplementary Table 4 Functional prediction of endometrial lavage microbiota in patients with EC/EH (n = 27) and control women (n = 8).

Supplementary Table 5 Microbial compositions at the species level in the following groups: 1) mid-vaginal microbiota of healthy non-pregnant women, 2) cervical microbiota of pregnant women, 3) endometrial lavage microbiota of women with benign endometrial lesions, and 4) endometrial lavage microbiota of women with EC/EH.

Supplementary Figure 1 Endometrial lavage samples analyzed in this study.
