# Supplementary material for: Analysis of endometrial lavage microbiota reveals an increased relative abundance of the plastic-degrading bacteria Bacillus pseudofirmus and Stenotrophomonas rhizophila in women with endometrial cancer/endometrial hyperplasia
Source: Front Cell Infect Microbiol. 2022 Nov 9;12:1031967. doi: 10.3389/fcimb.2022.1031967 (PMC9682088; doi:10.3389/fcimb.2022.1031967)

**Supplementary Figure 1.** Endometrial lavage samples and patients of this study. Sample identifiers were derived from **Supplementary Table 1**.


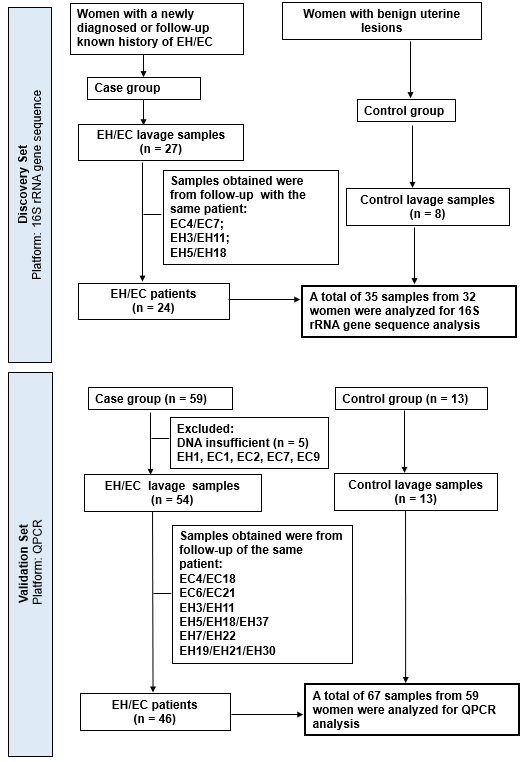

Supplement: Supplementary Figure 1 — Endometrial lavage samples analyzed in this study. [file Table_1.docx]
